# Updating search strategies for literature reviews with OUR2D2: an open-source computer application

**DOI:** 10.5195/jmla.2021.1105

**Published:** 2021-04-01

**Authors:** Abby M. Lohr, Noah Van Gorden, D. Jean McClelland, Ellen Dubinsky, Lynn B. Gerald, Ada Wilkinson-Lee, Scott C. Carvajal

**Affiliations:** 1 abbylohr@email.arizona.edu, Health Promotion Sciences Department, PhD Candidate, University of Arizona Mel and Enid Zuckerman College of Public Health; 2 nvangord@asu.edu, MCS Student, Arizona State University, School of Computing, Informatics, and Decision Systems Engineering; 3 jmcc@arizona.edu, Health Promotion Sciences Department, Program Director for Community Based Health Information Resources, University of Arizona, Mel and Enid Zuckerman College of Public Health; 4 edubinsky@arizona.edu, Office of Digital Innovation and Stewardship, Scholarly Communication Librarian, University of Arizona Libraries; 5 lgerald@arizona.edu, Health Promotion Sciences Department, Professor, University of Arizona, Mel and Enid Zuckerman College of Public Health; 6 adaw@arizona.edu, Department of Mexican American Studies, Associate Professor, University of Arizona, College of Social and Behavioral Sciences; 7 carvajal@arizona.edu, Health Promotion Sciences Department, Professor, University of Arizona, Mel and Enid Zuckerman College of Public Health

**Keywords:** literature search, open access, collaborative work

## Abstract

**Background:**

While writing a scoping review, we needed to update our search strategy. We wanted to capture articles generated by our additional search terms and articles published since our original search. Simultaneously, we strove to optimize project resources by not rescreening articles that had been captured in our original results.

**Case presentation:**

In response, we created Open Update Re-run Deduplicate (OUR2D2), a computer application that allows the user to compare search results from a variety of library databases. OUR2D2 supports extensible markup language (XML) files from EndNote and comma-separated values (CSV) files using article titles for comparisons. We conducted unit tests to ensure appropriate functionality as well as accurate data extraction and analysis. We tested OUR2D2 by comparing original and updated search results from PubMed, Embase, Clarivate Web of Science, CINAHL, Scopus, ProQuest Dissertation and Theses, and Lens and estimate that this application saved twenty-one hours of work during the screening process.

**Conclusions:**

OUR2D2 could be useful for individuals seeking to update literature review strategies across fields without rescreening articles from previous searches. Because the OUR2D2 source code is freely available with a permissive license, we recommend this application for researchers conducting literature reviews who need to update their search results over time, want a powerful and flexible analysis framework, and may not have access to paid subscription tools.

## BACKGROUND

In order to ensure maximum currency, a literature search will need to be repeated to capture any relevant articles that have been published or indexed during the many months typically necessary for the scholarly publication process to occur. Researchers have found that search strategies for literature reviews generally require significant time to develop, implement, and publish, including updates that may be required after manuscript submission and revision. In a survey of medical librarians conducting systematic reviews, respondents spent an average of 30.7 hours completing literature review tasks such as organizing meetings, developing the search strategy and translating it to other databases, documenting the process, delivering the results, and writing the methodology section of the manuscript, with the reported time spent on these core tasks ranging from 2 to 219 hours [1–3].

Due to the rapid pace of research and discovery, there is value in frequently and efficiently updating literature reviews. It is common for reviewers to ask authors to run their searches again before publication to ensure they have captured any new material as additional articles may change the authors’ conclusions [4]. Similarly, researchers may want to update published literature reviews to ensure that new evidence is incorporated into their findings [4]. This is especially critical in fields with quickly evolving medical treatments as well as quickly evolving health conditions. Finally, authors of living systematic reviews, an emerging type of literature review that is continuously updated, often revise their manuscripts on a monthly basis in an online-only format [5].

While writing a scoping review from 2018 to 2019 [6], we found ourselves in need of modifying our search and running it again. We wanted to capture articles generated by our additional search terms and articles published since our original search. Our goals were to update the search while also saving staff and student time and energy by not rescreening articles that had been captured in our original results. Throughout this manuscript, we will use the word “update” to refer to situations in which researchers adjust their search terms and/or conduct their searches again to identify new articles or gray literature. Additionally, we use “literature review” as an umbrella term to refer to articles that summarize existing knowledge such as scoping, systematic, narrative, and other reviews.

Throughout our scoping review project, we followed the Joanna Briggs Institute Reviewer's Manual, Chapter 11: Scoping Reviews, a set of scoping review guidelines that are increasingly used in health, applied, and social sciences. First, we conducted an initial search and fine-tuned our terms based on the results. Second, we ran our search in five databases. Third, we searched within the reference lists of identified articles for any additional articles that fit our inclusion criteria [7]. By the time we completed this work, including writing a full manuscript, twelve months had passed since the original search was conducted. At this point, we wanted to not only rerun our search but expand it to include new, related search terms. The development of a scoping review is an iterative process and allows for modification of the search strategy [7, 8].

Because manually comparing the results of a new search against an old search is tedious and time consuming, our aim was to fill the need for a clear, cost-effective, and reliable tool to update literature searches [9, 10]. In this case report, our objective was to present an open-source, nonproprietary tool entitled Open Update Re-run Deduplicate (OUR2D2) to compare two search results.

## CASE PRESENTATION

### Creating OUR2D2

**Figure 1:**
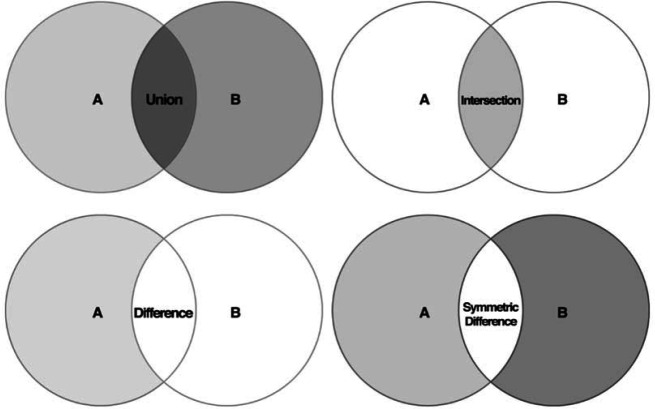
Set operation Venn diagrams [11]

We structured OUR2D2 using set operations. A set is a well-defined, distinct collection of objects such as a group of articles that meet a certain search criterion. OUR2D2 has four set operation options: (1) union: all articles combined, (2) difference: all articles that exist in only one file (in other words, subtract file A from B or vice versa), (3) symmetric difference: articles that are unique to both files, and (4) intersection: articles that are shared between both sets [11]. We describe the sets using Venn diagrams in Figure 1.

We included all four set operations as options within OUR2D2 to give users the ability to compare their search results in multiple ways. To look for the differences between old and new search results when running a search update, the difference set operation could be used. The union set operation could be used to combine multiple searches into one “master search results” comma-separated values (CSV) file and simultaneously display duplicates. Alternatively, to compare two searches in order to see articles that were not shared between either search, the symmetric difference set operation could be used, and those results could be verified with the intersection set operation. Regardless of the set operation selected, the summary box in OUR2D2 provides the cardinality (the count) of items resulting from each of the four set operations (Appendix A).

**Figure 2:**
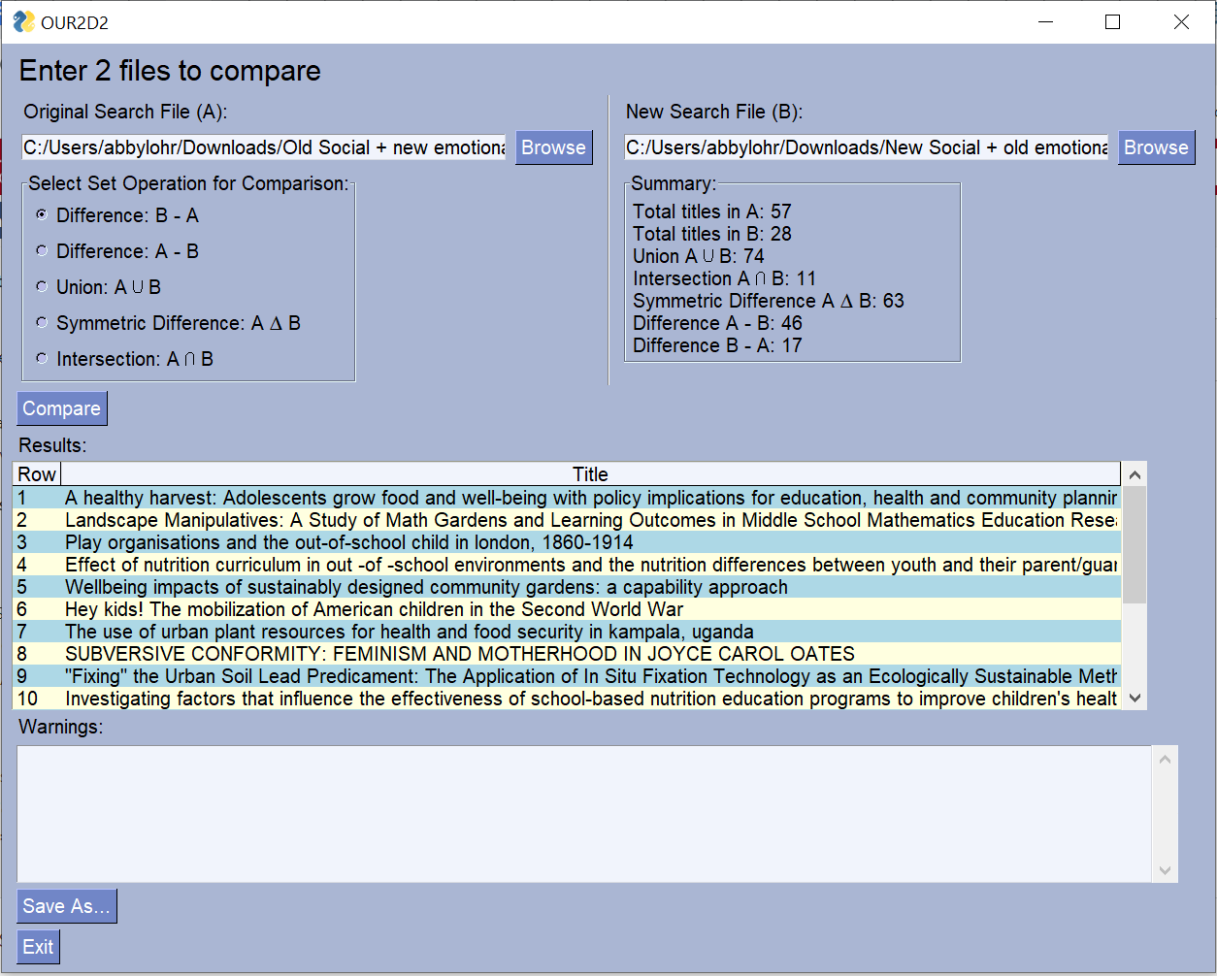
Screenshot of OUR2D2

OUR2D2 supports extensible markup language (XML) files from EndNote and CSV files with article titles listed in a column using any of the following labels: title, TI, or article title (Figure 2).

### Unit Testing OUR2D2

We tested OUR2D2 in fall 2019 and 2020. Using unit tests, we assessed OUR2D2 to assure appropriate functionality as well as accurate data extraction and analysis. Unit tests are automated analyses designed to examine whether a section of an application behaves as intended [12]. It is possible to write multiple unit tests for one section of code to ensure that all branches of logic are executed, and the desired outcome occurs with each test. Utilizing a unit testing framework called pytest [13], we tested the functions and logic in our source code. When a test resulted in failure, we identified the error and repaired the code to ensure that the test no longer failed. Additionally, we performed manual testing using CSV file formats that differed in character encodings, title column headings, and/or article titles. We tested the application with CSV files from the following databases: PubMed (legacy version), Embase, Clarivate Web of Science, CINAHL, Scopus, ProQuest Dissertation and Theses, and Lens (a freely available scholarly output database). Finally, we shared the application with an additional research librarian outside the project for further beta testing to ensure usability.

### Database Testing OUR2D2

In running our updated scoping review search, we expected many of the retrieved articles to be the same as those already screened. To enable a focus on newly identified literature and save time, we sought to avoid rescreening titles and abstracts that had been retrieved in the original search.

We used OUR2D2 to conduct two updated searches in each of our five chosen databases. In the first stage, we reran our original November/December 2018 search in October 2019 in each database, creating CSV files for the new results. Because we saved our original search results in an EndNote XML file, we used the difference set operation in OUR2D2 to compare this XML file to the new CSV file for each database. Across all five databases, we found 437 new articles. Overall, 378 remained after removing duplicates, 4 met titles and abstract inclusion criteria, and 0 met full text inclusion criteria. In the second stage, we updated our original search with new search terms, ran the new search, and compared the original and new search results. This update resulted in 19 new articles, of which 18 remained after removing duplicates, and 0 met title and abstract inclusion criteria. We did not need to clean the data before uploading it into the application, and OUR2D2 processed our results in seconds.

Using OUR2D2 saved our library-scientist team time. Before processing our second search results through OUR2D2 to remove articles we had already screened, we had 1,655 titles and abstracts to review after removing duplicates. If each title or abstract required 30 seconds on average to screen, the total time needed to review these titles and abstracts by our two reviewers would have been about 28 hours. Instead, we reviewed 396 new titles and abstracts (as described above) in about 7 hours. Thus, using OUR2D2 saved us 21 hours (10.5 hours per reviewer) of work rescreening articles.

## DISCUSSION

We recommend OUR2D2 for researchers conducting literature reviews who need to update their search results over time, want a powerful and flexible analysis framework, and may not have access to paid subscription tools. Additionally, this application could be helpful for individuals seeking to stay up-to-date on the literature in their field.

### Advantages: “Comparing apples from different trees”

OUR2D2 can compare searches based on article titles from paid subscription databases such as Clarivate Web of Science as well as freely accessible databases such as PubMed and Lens. By making OUR2D2 open source, anyone can access the tool and improve and build upon our code. We wrote the source code in the Python programming language, but users do not need knowledge of any computer programming language to use our custom application. The source code for the application is licensed under GNU Lesser General Public License version 3 (LPGLv3) and therefore freely available for download and modification. The GitHub repository contains a README.md file that has detailed instructions on usage of OUR2D2 [14].

Open-source software (OSS) and library cultural values are similar in that they have a desire to share ideas, expertise, and tools. Thus, it is beneficial for the two fields to work together [15]. Within library science, OSS can accelerate progress, channel innovation, increase library control over an application, and provide opportunities for customization [16]. Examples of collaboration between OSS and libraries include DSpace (an open source institutional repository platform) [17], Open Journal Systems (an open source journal publishing platform) [18], and FOLIO (an open source library services platform) [19].

The team that created OUR2D2 is another example of a synergistic collaboration between librarians and OSS developers. We worked together as codevelopers, following Eric Raymond's sixth lesson from *The Cathedral and the Bazaar*: “Treating your users as co-developers is your least-hassle route to rapid code improvement and effective debugging” [20]. As a result, our final product fit the needs of the library-scientist team and moved our scoping review forward.

The development of OUR2D2 has potential implications for future literature reviews. A literature review is a snapshot of knowledge available during the search process. If new knowledge is not included in a literature review before publication, the results may lack currency. For patients, this may mean that their healthcare providers make decisions without the latest information. Alternatively, it could cause researchers to pursue an area of study where new information is no longer needed [4].

OUR2D2 can help librarians by eliminating the task of rescreening titles and abstracts thereby saving them time. The time saved may vary based on the amount of material to be screened. While we estimate that OUR2D2 saved us twenty-one hours of work, this estimate should be interpreted cautiously as others have made similar, much smaller approximations [21]. As updating searches becomes more common, we encourage literature review authors to incorporate rerunning literature searches before final publication. By planning ahead and simplifying the procedure of comparing and deduplicating searches with tools like OUR2D2, researchers are better equipped for the iterative process of conducting a literature review.

## LIMITATIONS

OUR2D2 only compares articles by title. To do this, it removes all punctuation and changes all letters to lower case. We wrote the code in this way because across the databases we used, article title was the only consistent piece of exported information. However, each database used a different heading to label article titles (e.g., title, article title, or TI). Therefore, if an article title changes over time or is documented differently across databases, OUR2D2 will display false positives. For example, if an article includes special characters, OUR2D2 may mark these articles as different. In an article containing the word “β2-1,” EndNote converted the Greek letter to the word ‘beta’ while the CSV directly exported from PubMed kept the “β” in its original form. Consequently, it identified the article titles as unique. While a false positive is less of a concern than a false negative (an important article not identified), it is still important to carefully read through the results to ensure there are no duplicates of this nature. Thus, OUR2D2 does not entirely omit all screening but rather redundant screening. Human eyes are still needed to screen the OUR2D2 results. To make this process easier, we recommend comparing CSV files that are directly exported from the same database whenever possible. Additionally, if search terms include special characters, we suggest standardizing those characters in all CSV files before comparing search results.

Our article also has limitations in the context of existing literature on this topic. Although we present a free, easy-to-use application that was successful in the case of our scoping review, there are no definitive guidelines across disciplines on how to update a literature review search. Consequently, many methods have been published using EndNote [22], forward snowballing with Google Scholar [23], and visual text mining [9]. Until a standardized method is agreed upon, researchers will continue developing their own search update approaches and literature reviews will not be updated in a systematic way.

In this case report, we described the development of an open-source computer application called OUR2D2 to compare search results using set operations. OUR2D2 could be useful for librarians and researchers seeking to update literature review strategies across fields without rescreening articles from previous searches. We encourage expansion of our code and suggest that future software developers enhance the search capabilities by checking for unique characteristics that extend beyond title to detect duplicates, such as authors, coauthors, author networks, or Open Researcher and Contributor ID (ORCiD). Future research on the uses of OUR2D2 could include support for additional databases, improvement of the packaging of the code to create an installer for macOS, and exporting of additional data apart from article titles.

## Data Availability

Data associated with this article are available in Github: https://github.com/vangorden/OUR2D2.

## SUPPLEMENTAL FILES

**Appendix A:** OUR2D2 Instructions
