# Supplementary material for: Updating search strategies for literature reviews with OUR2D2: an open-source computer application
Source: J Med Libr Assoc. 2021 Apr 1;109(2):317–22. doi: 10.5195/jmla.2021.1105 (PMC8270383; doi:10.5195/jmla.2021.1105)
Supplement: Supplementary file 1 — Appendix A: OUR2D2 Instructions [file jmla-109-2-317-s01.docx]

Appendix A

**Open Update Re-run Deduplicate** (**OUR2D2) Instructions**

The technical requirements to use OUR2D2 are a computer with Internet access, Windows 10, and the permissions to install software from outside sources.

1. Download and open OUR2D2.
   1. Note: We advise taking caution when downloading any file from the Internet. Microsoft will warn you when downloading an executable file like OUR2D2 from the Internet. If you are concerned about the security implications of OUR2D2, the source code is freely available and you can compile it using PyInstaller (<https://www.pyinstaller.org/>) on your own device. We provided further instructions on how to do this in the README.md file (<https://github.com/vangorden/OUR2D2>).
2. Select two files to compare.
   1. When using Embase, be sure to select the “CSV fields by column” export option.
3. Upload the files into OUR2D2.
4. Choose a set operation to compare the two files. The first file is deemed set A and the second file is deemed set B. The set operations options are:
   1. Union: all the articles combined;
   2. Difference: all the articles that exist in only one file (subtracting: A-B or B-A);
   3. Symmetric difference: articles that are unique to both sets; and
   4. Intersection: articles that are shared between both sets.
5. Click on the compare button.
6. OUR2D2 will populate a table with a list of titles based on the set operation performed. If duplicates are found, OUR2D2 will produce a warning and display the duplicate titles on the screen (see Figure 2).
7. Users can then save the results to their computer in a CSV file and update their search results as needed.
